# Deepest and hottest hydrothermal activity in the Okinawa Trough: the Yokosuka site at Yaeyama Knoll

**DOI:** 10.1098/rsos.171570

**Published:** 2017-12-20

**Authors:** Junichi Miyazaki, Shinsuke Kawagucci, Akiko Makabe, Ayu Takahashi, Kazuya Kitada, Junji Torimoto, Yohei Matsui, Eiji Tasumi, Takazo Shibuya, Kentaro Nakamura, Shunsuke Horai, Shun Sato, Jun-ichiro Ishibashi, Hayato Kanzaki, Satoshi Nakagawa, Miho Hirai, Yoshihiro Takaki, Kyoko Okino, Hiromi Kayama Watanabe, Hidenori Kumagai, Chong Chen

**Affiliations:** 1 Department of Subsurface Geobiological Analysis and Research (D-SUGAR), 2-15 Natsushima-cho, Yokosuka 237-0061, Japan; 2 Research and Development Center for Submarine Resources, 2-15 Natsushima-cho, Yokosuka 237-0061, Japan; 3 Project Team for Development of New-generation Research Protocol for Submarine Resources, 2-15 Natsushima-cho, Yokosuka 237-0061, Japan; 4 Research and Development Center for Marine Biosciences, 2-15 Natsushima-cho, Yokosuka 237-0061, Japan; 5 Department of Marine Biodiversity Research (BIO-DIVE), Japan Agency for Marine-Earth Science and Technology (JAMSTEC), 2-15 Natsushima-cho, Yokosuka 237-0061, Japan; 6 Institute of Geochemistry and Petrology, ETH Zürich, Clausiusstrasse 25, 8092 Zürich, Switzerland; 7 Department of Systems Innovation, School of Engineering, The University of Tokyo, 7-3-1 Hongo, Bunkyo-ku, Tokyo 113-8656, Japan; 8 Department of Earth and Planetary Sciences, School of Science, Kyushu University, 744 Motooka, Nishi-ku, Fukuoka 819-0395, Japan; 9 Laboratory of Marine Environmental Microbiology, Division of Applied Biosciences, Graduate School of Agriculture, Kyoto University, Oiwake-cho, Kitashirakawa, Sakyo-ku, Kyoto 606-8502, Japan; 10 Atmosphere and Ocean Research Institute, The University of Tokyo, 5-1-5 Kashiwanoha, Kashiwa 277-8564, Japan

**Keywords:** biodiversity, chemosynthetic ecosystem, fluid chemistry, hydrothermal vent, microbial composition, sulfide deposit

## Abstract

Since the initial discovery of hydrothermal vents in 1977, these ‘extreme’ chemosynthetic systems have been a focus of interdisciplinary research. The Okinawa Trough (OT), located in the semi-enclosed East China Sea between the Eurasian continent and the Ryukyu arc, hosts more than 20 known vent sites but all within a relatively narrow depth range (600–1880 m). Depth is a significant factor in determining fluid temperature and chemistry, as well as biological composition. However, due to the narrow depth range of known sites, the actual influence of depth here has been poorly resolved. Here, the Yokosuka site (2190 m), the first OT vent exceeding 2000 m depth is reported. A highly active hydrothermal vent site centred around four active vent chimneys reaching 364°C in temperature, it is the hottest in the OT. Notable Cl depletion (130 mM) and both high H_2_ and CH_4_ concentrations (approx. 10 mM) probably result from subcritical phase separation and thermal decomposition of sedimentary organic matter. Microbiota and fauna were generally similar to other sites in the OT, although with some different characteristics. In terms of microbiota, the H_2_-rich vent fluids in Neuschwanstein chimney resulted in the dominance of hydrogenotrophic chemolithoautotrophs such as *Thioreductor* and *Desulfobacterium*. For fauna, the dominance of the deep-sea mussel *Bathymodiolus aduloides* is surprising given other nearby vent sites are usually dominated by *B. platifrons* and/or *B. japonicus*, and a sponge field in the periphery dominated by Poecilosclerida is unusual for OT vents. Our insights from the Yokosuka site implies that although the distribution of animal species may be linked to depth, the constraint is perhaps not water pressure and resulting chemical properties of the vent fluid but instead physical properties of the surrounding seawater. The potential significance of these preliminary results and prospect for future research on this unique site are discussed.

## 1. Introduction

The discovery of deep-sea hydrothermal vent [1,2] brought to light various unique processes occurring under the vast seawater mass. Four decades of cruise observations, in addition to theoretical and experimental approaches, led to the accumulation of a large dataset and knowledge with regards to global distribution of vent sites [3,4], heat and elemental flux between solid earth and ocean [5,6] including sulfide ore generation [7,8], physiological and phylogenetic characteristics of (hyper)thermophilic chemolithotrophic microbes [9,10] and vent-endemic fauna [11,12], as well as biogeography [13]. Based on this knowledge and from the primal perspective of energetics that bridges them [14], a generalized model predicting the relationships between underlying geology, fluid chemistry and microbial chemosynthetic primary production [15], also applicable to presume habitability of ancient Earth [16,17] and extraterrestrial bodies [18,19], has been built. Despite such significant progress in the understanding of each process and links among vent rock–fluid life, one obvious key question that has been raised since the initial discovery of vents [1] is yet poorly resolved today—that is the mechanisms and processes behind dispersal and thus realized distributions of vent-endemic organisms [20,21].

**Figure 1.**
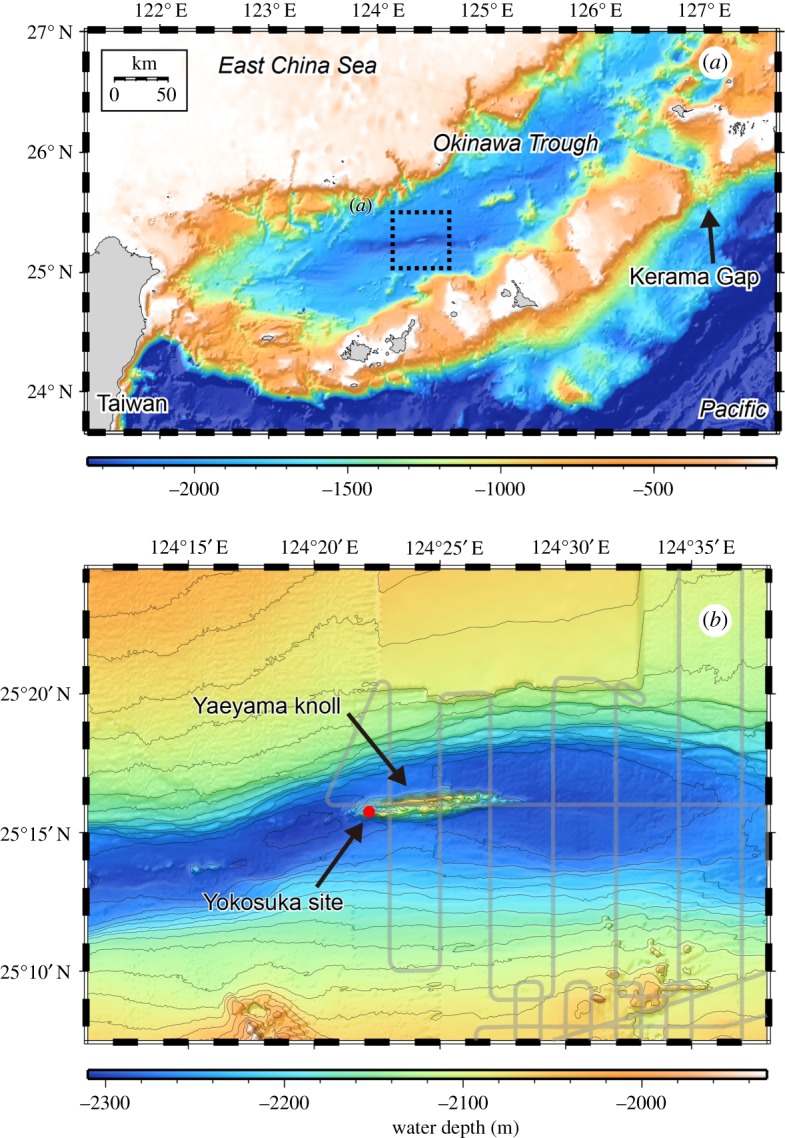
Seafloor topography of (*a*) entire southern Okinawa Trough and (*b*) Yaeyama Graben. Grey lines and a filled red circle in (*b*), respectively, represent surface ship track for geophysical observation including MBES-based hydrothermal site survey and the suggested location of hydrothermal activity. Seafloor topography in (*b*) was obtained by YK14-16, KR15-16 and YK16-07 cruises while partly sourced from a public database provided by Japan Coast Guard.

Understanding the mechanisms and factors regulating biodiversity and biogeography on the Earth is one of the most basic yet unresolved issues in biogeoscience. Findings from hydrothermal vent sites led to the confirmation of deep-sea chemosynthetic ecosystems, fuelled by chemical reactions between oxidizing components in seawater (e.g. O_2_, SO_4_) and reducing components originating at/beneath the seafloor (e.g. H_2_, CH_4_, H_2_S), often in the form of symbiosis [1,13,15]. Although it is now known that such ecosystems occur in other forms such as cold seeps and organic falls [22,23], hydrothermal vents and the surrounding ecosystems are most widely and systematically investigated at the global seafloor [13]. To date, more than 600 confirmed or inferred active vent sites are known [24], generally around plate boundaries. Vents on mid-oceanic ridges are distributed like stepping stones with intervals of a few to thousand kilometres between the neighbouring sites. Such systems pose significant difficulties in studying how biodiversity is formed within a biogeographic province [25], as the sites are distant and the dispersal is influenced by various factors from being in the open ocean. Indeed, such systems tend to have geographical barriers to dispersal and distribution of dominant animal species (e.g. [26]) within a region, making studying the influence of particular factors difficult. Vent sites within back-arc basins, on the other hand, tend to have well-mixed gene pools within basins and are well suited for investigating various factors governing biodiversity [21]. The East China Sea (ECS) is a semi-closed marginal sea between the Eurasian continent and the Ryukyu arc (figure 1*a*). The southeastern part of the ECS is characterized by an elongated, fault-controlled depression, that is called the Okinawa Trough (OT). The OT is an ideal study area for how the biodiversity within a site and biogeography of a species are regulated because of its topographic and oceanographic constraints. The ECS opens to the Pacific Ocean only via several straits shallower than 1100 m while its internal basin in the south, the southern OT (SOT), reaches depths greater than 2000 m (figure 1*a*). Despite only approximately 1300 km of the long axis (NE–SW direction), more than 20 hydrothermal vent sites have been detected here [27–29]. This close proximity among vent sites, in addition to the semi-enclosed oceanographic setting (e.g. [30]), provides high connectivity among each vent site within the OT [21].

Most of the OT vent sites investigated so far are located within a narrow range of water depth between 950 and 1880 m [27,31]. The predominant factor determining the fluid temperature is pressure, which depends on the seawater depth, and the temperature is, in turn, the significant factor determining the fluid chemistry [6]. Fluid chemistry is indeed similar among the OT sites, in general [32]. The narrow range of depths also allows prosperity of similar species which have physiological advantage against the pressure range around 10–15 MPa. In fact, research of microbes and vent fauna inhabiting OT hydrothermal sites in the past decades [33–35] have revealed a well-mixed genetic pool among the OT sites, except the two sites shallower than 900 m [36]. If a hydrothermal vent site is discovered at another distinct water depth range within the OT, it would provide insights to understand and evaluate the significance of depth, temperature, pressure and fluid chemistry in determining the biodiversity and biogeography. Such information is truly valuable towards effective conservation and management of vent resources and biodiversity in the light of possible upcoming anthropogenic exploitation of OT hydrothermal vents [37,38].

**Figure 2.**
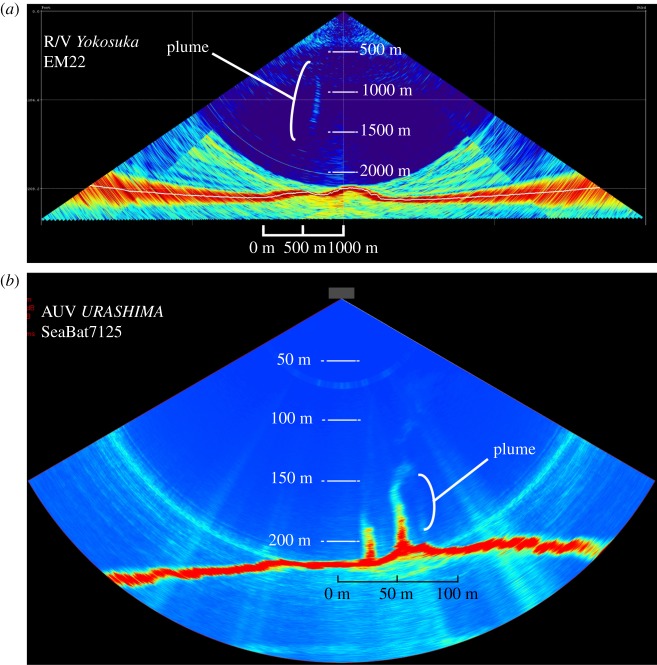
Images acquired by MBES equipped on (*a*) R/V *Yokosuka* and (*b*) AUV *URASHIMA*. Anomalous reflections elongating vertically in water column over the western end of the Yaeyama Knoll suggest some compounds which have physical properties distinct from seawater such as CO_2_ bubbles/hydrates and hydrothermal fluid.

**Figure 3.**
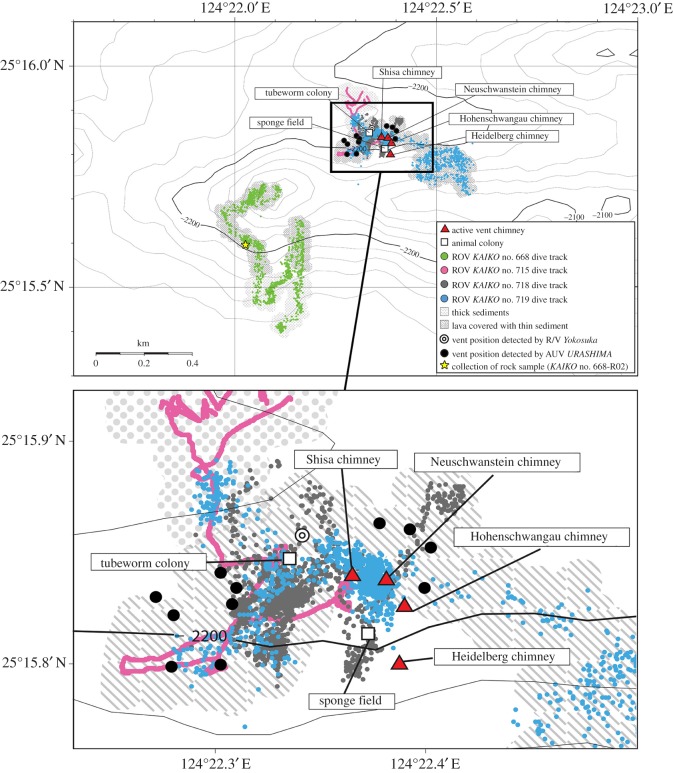
Event map for the Yokosuka site, large area map showing all relevant dive tracks is presented on the top with an expanded map of the hydrothermally active area (indicated by a solid box) on the bottom. Indications of each symbol are shown in the legend.

**Figure 4.**
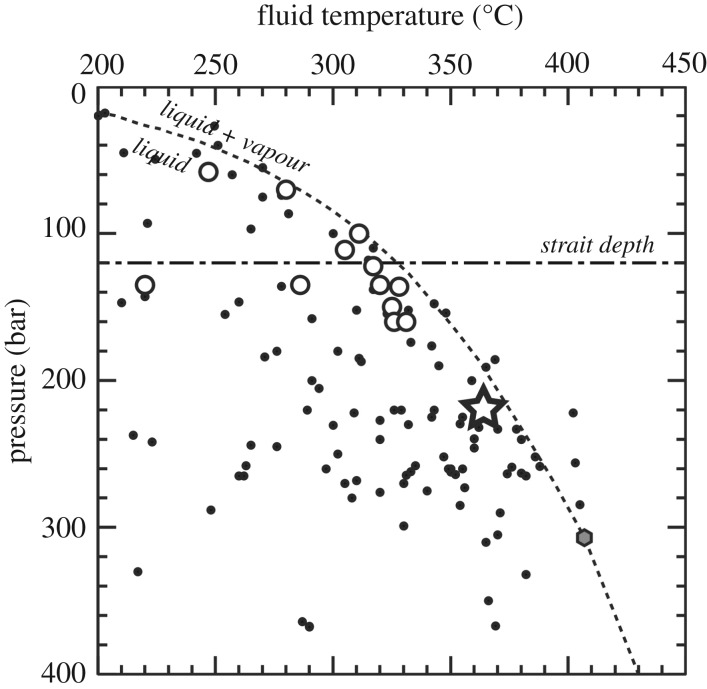
Pressure–temperature plot for the global hydrothermal sites (dataset used from [15,32]). An open star and open circles, respectively, represent the Yokosuka site and other OT sites, whereas black dots represent global hydrothermal sites. A broken curve and the grey hexagon, respectively, represent the two-phase boundary and critical point for 3.5% NaCl solution [44]. A horizontal broken-dot line represents the deepest strait in the East China Sea.

In this study, we surveyed the seafloor deeper than 2000 m in the SOT and discovered a hydrothermal fluid vent site at 2190 m depth, much deeper than the other known sites (approx. 1880 m) [39]. The existence of the present site was initially detected as an anomalous acoustic reflection of water column at the western end of the Yaeyama Knoll (figures 1*b* and 2); dives using the Remotely Operated Vehicle (ROV) *KAIKO* successfully located and surveyed the new vent site (figure 3). This new vent site is named ‘Yokosuka’ to honour the R/V *Yokosuka* which has contributed greatly to exploration of the global geofluid vent sites (e.g. [40–43]), including finding the initial signatures of the present site. The fluid of the Yokosuka site exhibited temperature up to 364°C, the highest recorded among all known OT sites (figure 4), the previous high temperature being 331°C recorded from the pNoho site (table 1). In the following, we outline the general picture of this new, hydrogeographically distinct, hydrothermal site in the OT with preliminary results from interdisciplinary studies across geochemistry, mineralogy, as well as microbiota and faunal composition.

**Table 1.** Endmember fluid chemistry of the Yokosuka site. Data from other Okinawa Trough sites and ambient seawater measured in this study are also presented for comparison.

region	southern OT	southern OT	southern OT	mid OT	mid OT	mid OT	southern OT
field	Yokosuka	Daiyon Yonaguni	Hatoma	Sakai	Izena	Iheya North	Yokosuka
site—vent	Hohenschwangau	Lion	C2	pNoho	Hakurei	Aki	ambient seawater
depth (m)	2182	1385		1600		1101	∼2180
MaxT (°C)	364	328	325	331	326	316	2
pH	5.4	5.7	5.4	4.7	4.7	4.45	7.4
Cl (mM)	153	614	303	678	608	594	563
Na (mM)	126	416	205	509	458	451	470
Na/Cl	0.82	0.68	0.68	0.75	0.75	0.76	0.83
K (mM)	8.6	86	40.8	99	75	82	9.6
K/Cl	0.06	0.140	0.135	0.146	0.123	0.138	0.017
Ca (mM)	4.3	23	11.4	27.0	23	22.3	9.6
Li (mM)	0.17		1.23	3.7	2.8	1.44	—
Si (mM)	10.2	11.3	8.63	13.7	11.3	14.1	—
B (mM)	4.05	3.9	2.33	4.4	3.7	2.09	0.54
Sr (mM)	0.024		0.046	0.111	0.13	0.086	0.087
Fe (mM)	<0.25	0.41		0.51		<0.3	—
Mn (mM)	0.6	1.25	417	1.01		0.76	—
NH_4_ (mM)	7.9	14.7	7.42	7.6	4.4	1.97	—
H_2_S (mM)	4.7		40.9	0.6		1.9	—
CO_2_ (mM)	294	22–329	(1770)	116	151	43–63	—
*δ*^13^C–CO_2_ (‰)	−8.2	−7.6	−8.2	−4.7	−6.2	−9	—
CH_4_ (mM)	9.6	1.2–13.5	10	3.2	6.8	0.4–0.9	—
*δ*^13^C–CH_4_ (‰)	−24.8	−26	−49.1	−27.8	−32.1	−48.4	—
*δ*^2^H–CH_4_ (‰)	−104			−111	−113	−112	—
C_2_H_6_ (µM)	69		40	<0.5	2.5	<0.5	—
C_1_/C_2_	139		250	>6400	2720	>800	—
H_2_ (mM)	9.43	0.8–5.5	1.2	0.35	1.4	0.03	—
*δ*^2^H–H_2_ (‰)	−369			−359	−379	−373	—
CO (µM)	71			<3	63	<0.5	—
*δ*^2^H–H_2_O (‰)	+2.4		−4.8		−0.6	−1.4	−0.43
*δ*^18^O–H_2_O (‰)	+1.7		1.1		1.6	1.3	−0.25
ref.	this study	Suzuki [45]	Toki [46]	Miyazaki [31]	Ishibashi [47]	Miyazaki [31]	this study

## 2. Material and methods

### 2.1. Yaeyama Knoll at the southern Okinawa Trough

#### 2.1.1. Geological background

The SOT is characterized by active back-arc rifting of the eastern end of the Eurasian continental margin which forms a typical topographic feature, the Yaeyama Graben (figure 1*a*). The Yaeyama Knoll is a young volcanic ridge (less than 1 Ma) [48] located in association with the rifting at the center of the graben (figure 1*b*). The knoll is elongated in east–west direction, parallel to the graben, so volcanism is considered to be constrained by normal faults forming the rift structure. The knoll is about 200 m high, and the shallowest water depth is 2050 m. The western end of the knoll is branched, forming two minor ridges.

#### 2.1.2. Hydrological background

The water depth of the Yaeyama Graben reaches 2300 m, far deeper than the deepest surrounding strait of ECS, the Kerama Gap (approx. 1100 m). Seawater deeper than 1100 m in the SOT is upwelled into the lower thermocline below the overlaying Kuroshio Current [30] with turnovers around 4.7–9.4 years [49]. The deep-water ventilation may allow larvae of vent-endemic animals to migrate from/into shallower hydrothermal vent sites beyond hydrogeographical barriers.

#### 2.1.3. Cruises and dives

Water column observation using acoustic devices such as multibeam echo sounder (MBES) and side scan sonar systems have been successfully applied to exploration of seafloor hydrothermal vents in the last decade [28,29,49–52]. Particularly, the MBES survey from the sea-surface is far more effective in detecting and roughly locating undiscovered hydrothermal fluid venting sites because of extensive seafloor coverage [28] when compared to conventional hydrocast survey [4,53]. During the R/V *Yokosuka* cruise YK14–16 in 2014, the MBES system (EM122, 12 kHz) detected a reflection anomaly in its water column records that arose vertically from the seafloor, suggesting hydrothermal activity, at the southern minor ridge at the western end of the Yaeyama Knoll (figures 1*b* and 2*a*). During the YK16–07 cruise and a dive of the Autonomous Underwater Vehicle (AUV) *URASHIMA* (dive no. 252) in 2016, acoustic anomalies were also detected by the AUV-based MBES (SeaBat7125, 200 kHz) (figure 2*b*) and side scan sonar (EdgeTech2200, 120 kHz) above the seafloor of the northern slope of the minor ridge (figure 3). Based on these localization data, deep dives using the ROV *KAIKO* (with vehicle *KAIKO Mk-IV*) were conducted during R/V *Kairei* cruises KR15-16 (dive no. 668) in 2015 and KR16-16 (dive nos. 715, 718, 719) in 2016 (figure 3). The Yokosuka site was successfully discovered on dive no. 715.

### 2.2. Sampling and analyses

#### 2.2.1. Fluid and rock chemistry

High-temperature hydrothermal fluids emerging from the chimneys were collected during ROV dives by a gas-tight fluid sampler, the WHATS-3 [31], in addition to ambient seawater collected by Niskin samplers as references. The full-fluid sample list is provided in electronic supplementary material, table S1. Fluid sampling and chemical analyses of the fluids collected followed procedures previously published (e.g. [31,54]). Fluid pH, alkalinity, NH_4_ concentrations and H_2_S concentrations were determined by a pH meter with titration and colorimetry [55]. Concentrations of Mg, Na, K, Ca, Sr, Si, B, Li, Fe, Mn, Zn, Cl and SO_4_ were determined with inductively coupled plasma optical emission spectrometry (ICP-OES: SPS5510; Hitachi High-Tech Science Corporation) and/or ion chromatography (IC: Dionex ICS-2100 and ICS-1600; Thermo Fisher Scientific) after appropriate sample dilution using Milli-Q deionized water (typically 200- to 2000-fold). Dissolved gases in the fluid samples were extracted on-board with a vacuum line, and concentrations of H_2_, CH_4_, CO, CO_2_ and C_2_H_6_ were determined onshore by gas chromatography with a helium ionization detector (GC-HID: GC4000, GL Sciences) and a flame ionization detector. Stable isotope ratios of CO_2_, CH_4_, CO, H_2_ and H_2_O were determined by continuous-flow isotope ratio mass spectrometry and a liquid water isotope analyser (Los Gatos Research, Inc.) and reported with delta (*δ*) notation to express the relative difference between minor/major isotope ratio (*R*) of a sample and the international standards (VSMOW for hydrogen and oxygen or VPDB for carbon) (*δ* = [(*R*_sample_/*R*_standard_) − 1]) in the permil scale [56].

A massive volcanic rock collected at non-hydrothermal seafloor was crushed to obtain unaltered inner-part fragments for subsequent chemical analyses. The fragments were washed with Milli-Q water in an ultrasonic bath and then further crushed to powder form using tungsten carbide and agate mills. Abundances of major and trace elements were determined by X-ray fluorescence analysis and ICP-quadruple mass spectrometry, respectively, using procedures reported previously [57].

#### 2.2.2. Microbiological analyses

A single chimney structure (1300–11 600** **g) was collected from each of the three vents, the Neuschwanstein, Hohenschwangau and Heidelberg vents (figure 3), for microbiology. Immediately after the recovery on-board, samples were stored at −80°C until use. To estimate the abundance of microbial cells, chimney samples (0.8–1.6** **g) were fixed with 3.7% formaldehyde-phosphate buffered saline (PBS) for 2 h at 4°C. After washing with PBS three times, samples were sonicated on ice with eight 5 s pulses with VP-050 ultrasonic homogenizer (power, 20%: TAITEC, Koshigaya, Japan) with 5 s pauses between power pulses. Suspended cells were filtered on 0.2** **µm pore size Isopore filters (Merck Millipore, Schwalbach, Germany), and stained with SYBR Gold (Thermo Fisher Scientific, Waltham, MA, USA) for 10** **min. After washing with PBS, microbial cells were counted in triplicate with an ECLIPSE Ni epifluorescence microscope (Nikon, Tokyo, Japan).

For DNA extractions, chimney samples were pulverized with a mortar and pestle in liquid nitrogen. DNA was extracted from each chimney sample (2.7–4.6** **g) using the TRIzol reagent (Thermo Fisher Scientific) as described elsewhere [58]. V4-V5 regions of the 16S rRNA gene were amplified and analysed with a MiSeq sequencer (Illumina, San Diego, CA, USA) as previously described [59]. Sequences were processed using the QIIME software package [60]. Operational taxonomic units (OTUs) were selected based on 97% similarity level using the UCLUST [61] and were assigned to a reference taxonomic classification using the SILVA 119 [62] and the RDP classifier (SSU ref NR 119; http://www.arb-silva.de/no_cache/download/ archive/release_119/Exports). The raw sequence data have been deposited in GenBank/EMBL/DDBJ with the accession no. DRA005734.

#### 2.2.3. Identification and relative abundance of megabenthos

High-quality video and images were taken using the camera system of ROV *KAIKO* during the deep dives, which allowed identification of megabenthos present in various areas of the Yokosuka site. Where possible, faunal specimens were collected using the manipulator or a slurp gun with a single-chambered sample chamber, equipped on the ROV. Upon recovery on-board R/V *Kairei*, the specimens were sorted and identified morphologically to ground-truth the video/photo-based identifications. Occurrences of each animal species were noted for various areas visited throughout the dives by analysing all available video and images for the areas of interest (i.e. active chimneys and peripheral faunal assemblages), and their relative abundances were estimated using dominant–abundant–common–occasional categories (as employed in [63]).

To investigate the extent of similarity in megabenthos species composition among different habitats within the Yokosuka site, Jaccard's index of similarity was calculated based on presence/absence data of the taxa identified. Nonmetric multidimensional scaling (nMDS) was used to visualize the data, and similarity contours based on a group-average clustering analysis was overlaid on the nMDS plot to indicate the extent of faunal resemblance among the closely clustering habitats (after [36]).

## 3. Results and discussion

### 3.1. Overview of the Yokosuka site

Figure 3 is a summary of all dive tracks, mapped against the newly discovered chimneys, and other indications of hydrothermal activity. During ROV *KAIKO* dive no. 668, the southern slope of the southern minor ridge was surveyed for hydrothermal signatures such as increases in water column turbidity and benthic animal density, but none was detected. A rock sample (*KAIKO* no. 668-R02; figure 3) was collected to characterize the volcanic body, the chemical analysis of which revealed K-medium basaltic andesite (island arc tholeiite; electronic supplementary material, table S1) standing on a mafic endmember of the bimodal composition of OT volcanic rocks [64]. Some other OT hydrothermal activities, on the other hand, are hosted by another endmember, dacite to rhyolite (e.g. [29,45,65]).

**Figure 5.**
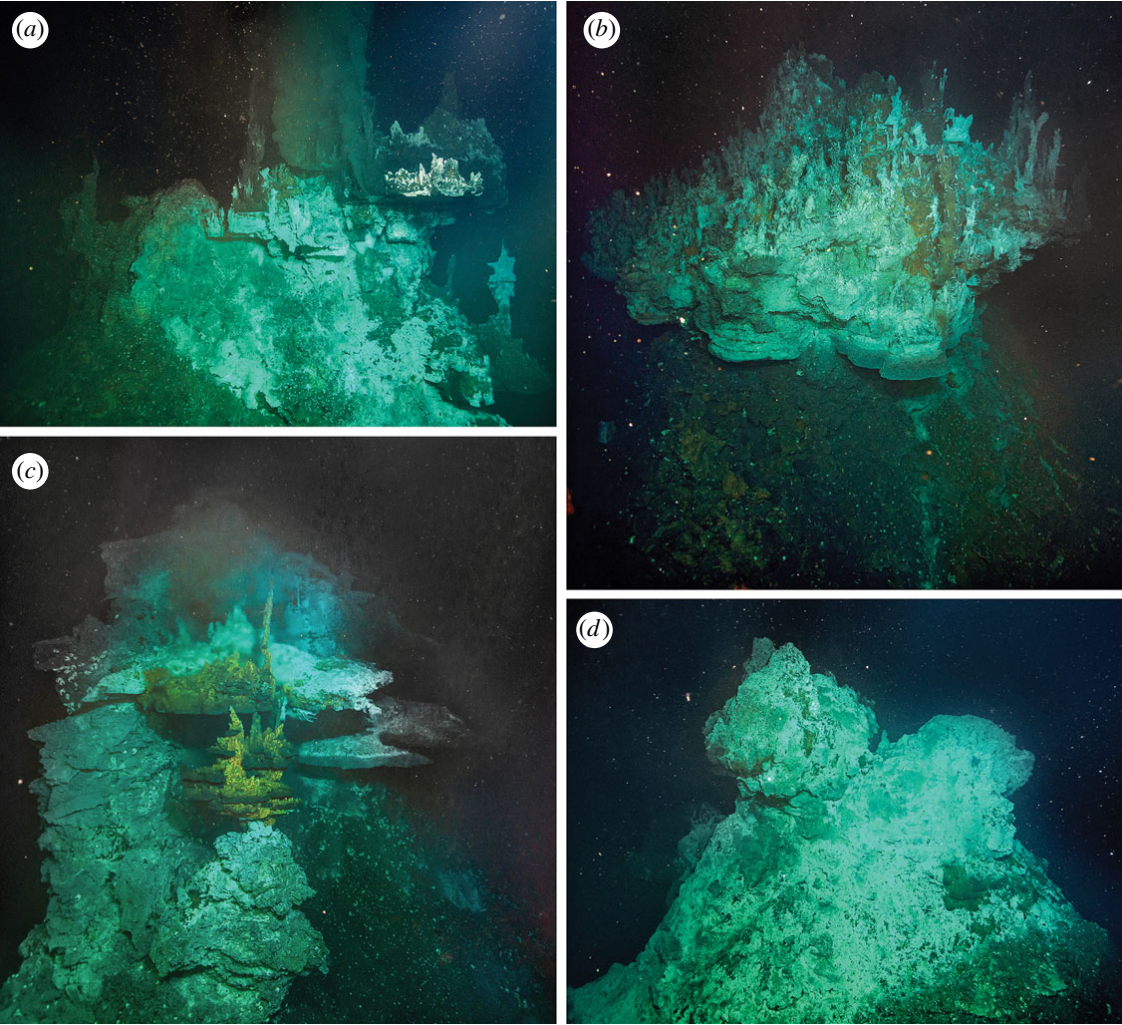
Major vent chimneys in the Yokosuka site. (*a*) Neuschwanstein chimney (max temp. = 356.9°C), (*b*) Hohenschwangau chimney (max temp. = 364.1°C), (*c*) Heidelberg chimney (max temp. = 349.9°C) and (*d*) Shisa chimney.

**Figure 6.**
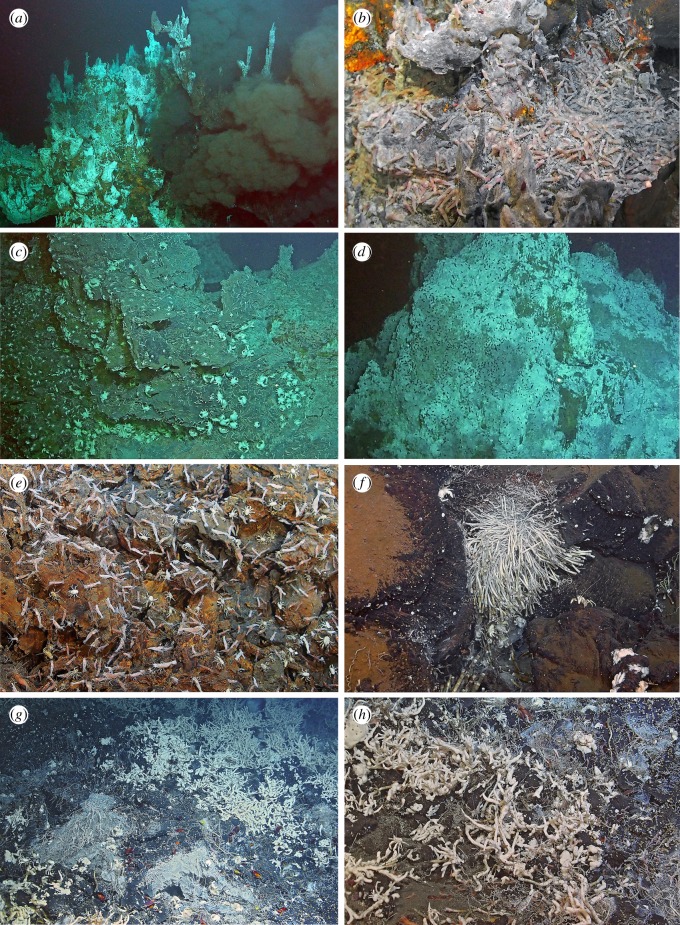
Megafaunal communities of the Yokosuka site. (*a*) Overview of shrimp aggregations on the top of the Neuschwanstein chimney. (*b*) Close-up of a *Shinkaicaris leurokolos* alvinocaridid shrimp aggregation on the Hohenschwangau chimney. (*c*) *Shinkaia crosnieri* squat lobsters near the base of the Hohenschwangau chimney. (*d*) Large aggregations of scale worms (black dots) on the surface of the Shisa chimney. (*e*) A typical animal colony around the base of chimneys of the Yokosuka vent field dominated by *Alvinocaris longirostris* shrimps, *Munidopsis ryukyuensis* squat lobsters, and *Provanna clathrata* snails. (*f*) Peripheral tubeworm (*Lamellibrachia* sp. and *Alaysia* sp.) bush 50 m east of the Neuschwanstein chimney. (*g*) Overview of a peripheral community visually dominated by poecilosclerid sponges near the Heidelberg chimney. (*h*) Close-up of the sponge-dominated peripheral community, most notable animals being *Lamellibrachia* sp. and *Alaysia* sp. tubeworms and *B. aduloides* mussels.

On *KAIKO* dive no. 715, the ROV landed at a depth of 2178 m on the northern slope of the minor ridge, where the seafloor was covered with thick sediment. After travelling southwards for about 150 m we arrived at a rocky field with thin sediment cover and occasionally with whip corals and sponges growing. As we travelled further south, water became increasingly turbid and corals disappeared, being replaced by rossellid sponges, commonly seen around other OT hydrothermal sites [66], and we soon encountered a colony of tubeworms sustained by diffuse flow venting (depth 2205** **m). After travelling about 40 m eastwards, we then encountered rusty microbial mats, a field of small pieces of broken sulfide, and further animals typical of vents, such as the squat lobster *Munidopsis* spp. and vent shrimps *Alvinocaris*. Another 10 m eastwards, we discovered a very active black-smoker chimney with large flanges (approx. 12 m tall, depth 2190 m at base; figures 5*a* and 6*a*). We named this chimney **‘**Neuschwanstein**’** and collected animals, chimney and hydrothermal fluid. The highest fluid temperature measured was 347.3°C, the highest ever recorded at this point in all of OT vents [32]. However, we were unable to collect sufficient numbers of hydrothermal fluid sample (only one of four available bottles of WHATS-3 sampler [31]) because fragile flanges filled with high-temperature fluids prevented us from approaching close to the venting orifices, for safety reasons. With poor visibility and unstable positioning signals, we had difficulty identifying the absolute position of the ROV during this dive. In particular, the position shown by INS (Inertia Navigation System) and SSBL (Super Short Base-Line) were in disagreement.

On dive no. 718, the ROV landed northeast of the best-estimated position of the Neuschwanstein chimney (figure 3). The seafloor was mostly sediment-covered, with occasional rossellid sponges. After exploring the vicinity against poor visibility for about an hour, we encountered a field of broken sulfide pieces inhabited by squat lobsters, similar to that seen near Neuschwanstein. Climbing a mound nearby the sulfide field revealed a large, mushroom-like black-smoker chimney with many flange structures. This chimney was obviously distinct from Neuschwanstein, and we named it ‘Hohenschwangau’ (approx. 7 m tall, depth 2190 m at base, figure 5*b*). Sampling of animals, chimney fragments and hydrothermal fluid was carried out; the highest temperature recorded was 364.1°C, even higher than Neuschwanstein and close to the boiling point at its depth (figure 4) [44]. After sampling and travelling northwest, we located a spot of shimmering water about 20 m away from Hohenschwangau. Further exploring the area, we travelled through an area of brown discoloration, apparently old dead chimneys covered by small sponges (as is typical for OT vent periphery areas), and finally, back to rocky seafloor. Then, to the southwest of Hohenschwangau, a very large area of diffuse flow venting appeared (figure 6*g*). This area was visually dominated by a branching demosponge in the order Poecilosclerida (similar to previously reported from the Minami-Ensei vent site) [66]. The occurrence of this type of sponge field here is intriguing as the Minami-Ensei site is a very shallow site (Depression B where poecilosclerid sponges were common is around 700 m deep [67]). We then moved southeast for about 30 m, where we discovered a further large mound-like chimney with large flange structures. This was clearly different from either Neuschwanstein or Hohenschwangau, and we named it **‘**Heidelberg**’** (approx. 10 m tall, depth 2165 m at base, figure 5*c*). Again we carried out sampling of animals, chimney pieces and hydrothermal fluids; the highest recorded temperature was 349.9°C.

For the final dive at the Yokosuka site (no. 719), the ROV landed on sediment-covered seafloor southwest of Neuschwanstein. After travelling for about 100 m northeast, we encountered sediments with brown discoloured mat followed shortly by white microbial mat and broken sulfide pieces. Continuing along the same direction, we soon found a large mound-like chimney that only had concentrated venting at a bulge-like flange structure at the side. We gave it a name, **‘**Shisa**’** (approx. 10 m tall, depth at base 2188 m, figure 5*d*), but no sampling was carried out. Carrying on the same direction past Shisa, we at last rediscovered Neuschwanstein chimney and commenced hydrothermal fluid sampling (maximum temperature 356.9°C). Although we further travelled eastward up to 400 m away from the four discovered chimneys, the seafloor was consistently rocky with occasional sponges after leaving Neuschwanstein and no further signs of hydrothermal activity were found.

### 3.2. Fluid chemistry

**Figure 7.**
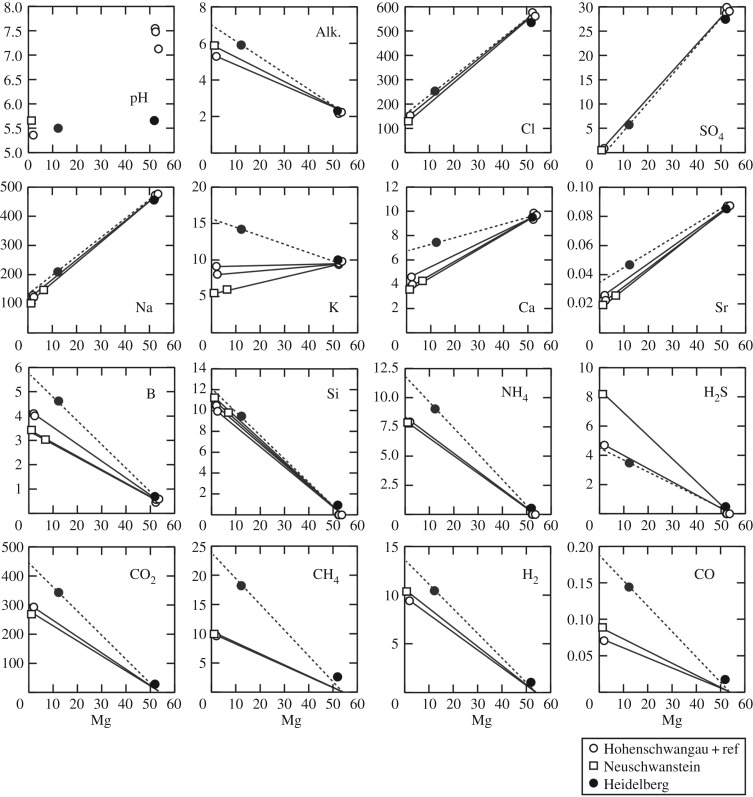
Mg diagrams for Yokosuka site fluids. Open circles represent the chemical composition of the Hohenschwangau fluids and ambient seawater. The Neuschwanstein and Heidelberg fluids are shown by open squares and filled circles, respectively. Connecting lines from ambient seawater to each low-Mg fluid (dotted line for Neuschwanstein fluid) represent their extrapolation to Mg = 0 for estimating endmember fluid composition. All the concentrations are presented in the unit of millimolar.

Concentrations of each chemical species dissolved in the fluid samples are shown in the magnesium diagram (figure 7) and all analytical results are listed in electronic supplementary material, table S2. The fluid pH of samples, with low magnesium concentrations, ranged between 5.3 and 5.7. Cl and Na concentrations of the low-Mg fluids were significantly lower than those of the ambient seawater (figure 7), being approximately one-third of their seawater values. As Cl has few removal processes during subseafloor fluid circulation, the result is interpreted to be due to subcritical phase separation (boiling) (figure 4) and preferential emergence of the resulting vapour-rich phase fluid (e.g. [68,69]). Fluids showing Cl enrichment compared with the seawater level were not found within the Yokosuka site, although simultaneous venting of vapour-rich and -depleted phases at distinct localities within a hydrothermal field has been found (e.g. [45,47,70]).

Mg diagrams of each chemical species did not display a single mixing line from Mg-rich ambient seawater to Mg-depleted endmember fluid composition (figure 7), suggesting intra-field (inter-chimney) variation of the venting fluid chemistry. The estimated endmember fluid of the Neuschwanstein chimney contains relatively abundant ions, approximately 1.5 times higher than those of Heidelberg and Hohenschwangau chimneys. Inter-chimney differences in fluid chemistry probably result from differences in magnitudes and patterns of the two-phase segregation, due to uniform Na/Cl and K/Cl ratios among chimneys (table 1). Nevertheless, even in the Neuschwanstein fluid, estimated endmember concentrations of ion species (Cl, Na, K, Ca, Sr, Li, etc.) were the lowest values among those observed so far among all OT hydrothermal sites (table 1).

The endmember K/Cl value, which is a representation of the K content in the primitive upwelling fluid without influence from the phase separation, is significantly lower in the Yokosuka site fluids (0.055) than fluids of other OT sites (greater than 0.09) [32,46]. As the fluid K content is dominated by that of the host rock, which interacts with fluid flowing in the subseafloor, the low K/Cl value of the Yokosuka fluid points to a low K_2_O in the host rock. This seems to be consistent with basaltic andesite and its low K_2_O content (0.9%) exhibited by the Yaeyama Knoll body, which is probably identical to the subseafloor host rock of the Yokosuka site.

**Figure 8.**
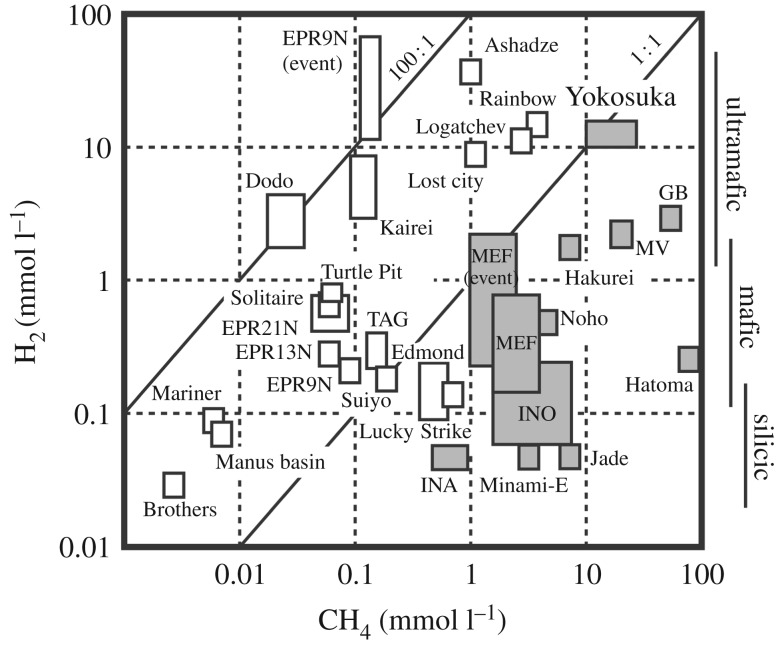
A plot of H_2_ and CH_4_ concentrations in high-temperature hydrothermal fluids. Both axes are shown with a logarithmic scale. Grey-coloured and open squares, respectively, represent sediment-involved and sediment-not-involved hydrothermal sites. After Kawagucci *et al.* [54] with modification and additional data shown in this study (table 1).

Each of the volatile species dissolved in the endmember fluid was notably abundant (table 1). The endmember fluid of the Neuschwanstein chimney contained much more volatiles, except H_2_S, than the other two chimneys (figure 7). Though the high volatile concentrations are mostly attributable to preferential venting of the vapour-enriched phase through subseafloor fluid boiling under subcritical condition [71] (suggested by the depletion of Cl), fluid–sediment interaction is also expected to generate and contribute some volatiles. The Yokosuka site hydrothermal fluids contained CH_4_ and H_2_ both as high as 10 mM, the first example among all global hydrothermal sites investigated so far [54,72] (figure 8). The high concentrations of CH_4_ and H_2_, comparable to H_2_S (up to 8.2 mM), potentially provide large yields of bioavailable energy for hydrogenotrophic and methanotrophic metabolisms in the mixing zone [15]. Relative abundance of methane against ethane (C_1_/C_2_ ratio) ranges 75–140, lower than typical range in the OT fields (approx. 10^3^ [73]).

The abundance of CH_4_ in sediment-associated hydrothermal sites, including the Yokosuka site, is thought to result from the thermal decomposition of sedimentary organic matter and microbial methanogenesis in the anoxic sediment [73,74]. The *δ*^13^C–CH_4_ value of −25.7‰ of the Yokosuka fluid is the uppermost value in the *δ*^13^C–CH_4_ range observed in the sediment-associated sites (−58 to −25‰) and suggests a predominance of thermogenic CH_4_ rather than biogenic one. The C_1_/C_2_ ratio supports the thermogenic origin of methane. As abundant H_2_ exceeding 1 mM is only seen with ultramafic-rock- and fresh basalt-hosted hydrothermal activities [54,68,72], the basaltic andesite-hosted activity of the Yokosuka site probably constrains H_2_ and CH_4_ concentrations to as low as 0.1 mM in the subseafloor fluid reservoir (figure 8). As thermal interaction of sediment beneath the vent can provide additional H_2_ to the upwelling fluid [47], thermogenic H_2_ input prior to phase separation would be able to account for the abundant H_2_ observed. The *δ*^2^H values of CH_4_ (−110‰) and H_2_ (−356‰) are in accordance with the hydrogen isotope equilibrium with H_2_O having *δ*^2^H–H_2_O of +0‰ at the endmember fluid temperature of 364°C [75]. The lower *δ*^2^H–H_2_ value in the Neuschwanstein chimney fluid, containing significant sulfate (no. 719-W1), may be attributable to microbial H_2_ oxidation after fluid cooling in the sampler bottle. Previously, hydrogenotrophic sulfate reducers, in particular, were reported to decrease the *δ*^2^H–H_2_ value approximately below −600‰ [76,77]. Indeed, microbiological analysis revealed hydrogenotrophic sulfate-reducing microbes inhabiting the Neuschwanstein chimney (see the next section), supporting this hypothesis.

The CO_2_ concentration of Yokosuka site fluids (approx. 300 mM) was as high as vent fluids from other SOT hydrothermal sites (table 1). Although magma degassing is presumed to be the initial source of abundant CO_2_ in the subseafloor fluid reservoir of back-arc hydrothermal sites (e.g. [70,78]), the *δ*^13^C–CO_2_ values varied among the chimneys (−11 to −6‰). Inputs of thermogenic CO_2_ with *δ*^13^C–CO_2_ of approximately 25‰ after branching of the upwelling fluid into each chimney, in addition to the primitive CO_2_ having *δ*^13^C–CO_2_ of −6 to 0‰ [79], may be accountable for the inter-chimney *δ*^13^C–CO_2_ variation in the Yokosuka site. The isotope effect on phase separation is another possible source of this variation, but it seems inconsistent with small variations in isotope ratios exhibited by other volatiles. Microbial production/consumption is not considered to be an attributable source due to the lack of carbon species comparable with CO_2_, from a stoichiometric viewpoint.

### 3.3. Microbial composition

The total microbial cell counts (mean ± standard deviation) in chimneys from Neuschwanstein, Hohenschwangau and Heidelberg vents were 2.3 × 10^7^ ± 2.9 × 10^6^ cells g^−1^, 1.2 × 10^6^ ± 2.0 × 10^5^ cells g^−1^ and 1.4 × 10^7^ ± 3.3 × 10^6^ cells g^−1^, respectively. These cell abundances were similar with those previously reported from other deep-sea vents (10^5^–10^8^ cells g^−1^ [80–83]), despite the notable enrichment of energetic molecules in the fluid.

**Figure 9.**
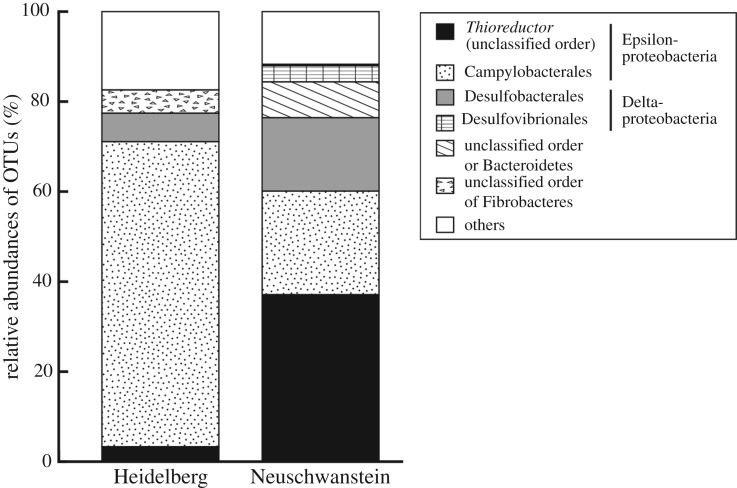
Composition of the microbial community based on taxonomic grouping (order level) of 16S rRNA gene amplicon reads. DNA was extracted from a single pulverized chimney sample from each vent. OTUs with greater than 3% frequency in either sample are presented, and the rest and unassigned taxa are indicated as ‘others’.

Microbial community structures were assessed via 16S rRNA gene amplicon sequencing. The PCR amplicon was not obtained from Hohenschwangau chimney, which had the lowest cell density. A total of 1169 different OTUs were identified from Neuschwanstein and Heidelberg chimneys on the basis of classification with greater than or equal to 97% of identity. Although the proportions of archaeal reads were low (1.3% and 1.2% in Neuschwanstein and Heidelberg, respectively), Methanococcales and Thermoplasmatales members were the most frequently detected archaea in Neuschwanstein and Heidelberg, respectively. Members of the order Methanococcales are hydrogenotrophic methanogens widely distributed in the OT hydrothermal fields [81,84,85]. The most abundant phylum was Proteobacteria in both chimney structures; 81.1% and 80.3% of reads in Neuschwanstein and Heidelberg, respectively. At the class level, the microbial communities were dominated by the phylotypes of Epsilonproteobacteria (60.1% and 71.1% in Neuschwanstein and Heidelberg, respectively), and were followed by the phylotypes of Deltaproteobacteria (16.3% and 6.3% in Neuschwanstein and Heidelberg, respectively) (figure 9). Members of the class Epsilonproteobacteria represent common and prevalent microorganisms in deep-sea vents of various depths [86]. Most of the known deep-sea vent Epsilonproteobacteria are strict chemoautotrophs using hydrogen and/or sulfur compounds as electron donors and nitrate, oxygen and sulfur compounds as electron acceptors [87]. Among Epsilonproteobacteria, members of the genus *Thioreductor* were frequently detected in the Neuschwanstein chimney (figure 9). In contrast, members of the genera *Sulfurovum* and *Sulfurimonas* (order Campylobacterales of the class Epsilonproteobacteria) were abundant in the Heidelberg chimney. All of these Epsilonproteobacteria are mesophilic chemoautotrophs [87]; however, *Thioreductor* species lack the ability to use sulfur compounds as the energy source and instead uses H_2_ as the primary energy source [80]. In previous studies in the OT hydrothermal fields, members of *Thioreductor* had a relatively limited distribution compared with those of *Sulfurovum* and *Sulfurimonas* [35,81,84,85]. For example, in the Iheya North Original hydrothermal site (water depth = approximately 1000 m; maximum fluid temperature = 311°C), members of Campylobacterales were frequently detected in the vicinity of deep-sea hydrothermal vents. By contrast, members of the genus *Thioreductor* were dominantly detected only in the *in situ* cultivation device deployed into the gas-rich fluid flow [84]. Similarly, hydrogenotrophic sulfate-reducing bacteria, e.g. *Desulfobacterium* species of the class Deltaproteobacteria, were abundantly detected only in Neuschwanstein chimney. These suggested that more H_2_-rich vent fluids in Neuschwanstein resulted in the dominance of hydrogenotrophic chemolithoautotrophs within the associated chimney structure.

### 3.4. Megabenthos composition

In the hydrothermally active areas of the Yokosuka vent site, 21 species of megabenthos were identified (table 2), including two demosponges common in the periphery, four gastropods, one bivalve, six annelids including two tubeworms, and eight crustaceans including three alvinocaridid shrimps. All were species already known to be present in OT hydrothermal vents [36], although some could not be identified to species level (such as the poecilosclerid sponge) and a few still await formal taxonomic description (such as *Alvinocaris* sp. *sensu* [34]). The vent field could be divided into a few visually distinct regions differing in the dominant taxa and species present, including surface of active chimneys, base of active chimneys, diffuse flow sites dominated by tubeworm bushes, and periphery area dominated by poecilosclerid sponges. The species richness of the regions increased from focused vigorous venting towards weakly hydrothermally influenced peripheral areas (table 2).

**Table 2.** List of megafauna species inhabiting the Yokosuka site and variation in occurrence and relative abundance among various habitats according to dominant–abundant–common–occasional categories as employed previously [63].

		chimney surface						
group	taxa	Neuschwanstein	Hohenschwangau	Heidelberg	Shisa	chimney base	diffuse flow (tubeworm bush)	periphery (sponge field)
Porifera	Poecilosclerida indet.	−	−	−	−	−	−	++++
	Demospongiae indet.	−	−	−	−	+	+	++
Mollusca: Gastropoda	*Bathyacmaea* sp.	−	−	+	−	+	++	+
	*Lepetodrilus nux*	−	−	−	−	−	++	+++
	*Provanna clathrata*	−	−	+		+++	++	++
	*Provanna subglabra*	−	−	+	−	+	+	+
Mollusca: Bivalvia	*Bathymodiolus aduloides*	−	−	−	−	+	++	++
Annelida	*Lamellibrachia* sp.	−	−	−	−	−	++++	+++
	*Alaysia* sp.	−	−	−	−	−	+++	+++
	*Amphisamytha* sp.	−	+	+++	−	+	++	++
	*Paralvinella* aff. *hessleri*	+++	−	−	−	−	−	−
	*Branchinotogluma* sp.	+++	++	+	++++	−	−	−
	*Lepidonotopodium?* sp.	−	−	−	−	−	+	−
Arthropoda: Crustacea	*Shinkaicaris leurokolos*	++++	++++	+++	++	−	−	−
	*Alvinocaris longirostris*	++	+	+++	+++	++++	++	++
	*Alvinocaris* sp.	++	+	+++	+++	+++	++	++
	*Lebbeus shinkaiae*	−	−	−	−	+	+	++
	*Shinkaia crosnieri*	−	+	+++	−	+	++	++
	*Munidopsis ryukyuensis*	−	−	−	−	+++	++	++
	*Munidopsis longispinosa*	−	−	−	−	+	+	+
	*Neoverruca intermedia*	−	−	−	−	−	+	+

On the two most active chimneys (figure 6*a*), *Shinkaicaris leurokolos* shrimps, *Branchinotogluma* sp. scale worms and *Paralvinella* aff. *hessleri* polychaetes lived in closest proximity to high-temperature effluents (figure 6*b*), with all three being present on Neuschwanstein but *P.* aff. *hessleri* being absent in Hohenschwangau. These three species found to dominate the area close to vent effluent are known to be the most heat-tolerant of vent animals found in OT, and are usually found close to a vent orifice [34,88]. Further away from the fluid source, a high abundance of two species of *Alvinocaris* shrimps (*A. longirostris* and *A.* sp. *sensu* [34]) was observed. Some individuals of the squat lobster *Shinkaia crosnieri* were present in the lower part of the chimney in Hohenschwangau, although this was not the case in Neuschwanstein. This distribution reflects different tolerance levels of the animals and that *Alvinocaris* and *Shinkaia* are less able tolerate strong vent effluents [34,36] of the two black-smoker chimneys. A commensal polychaete, *Amphisamytha* sp., was found on the body surface of *S. crosnieri* across the vent field.

At Heidelberg chimney where venting was less robust, zonation was not as clear with the two species of *Alvinocaris* being equally abundant but with *S. crosnieri* being present at much higher abundance (figure 6*c*). *Paralvinella* was not present, and *Branchinotogluma* was present only in low abundance. Three more peripheral molluscs including two snails in genus *Provanna* and one limpet in genus *Bathyacmaea* were present too, albeit in low abundances (category ‘occasional’, table 2). The Shisa chimney is rather strange in that although it was not vigorously venting at all, its entire surface was nonetheless covered by a high abundance of *Branchinotogluma* sp. scale worms (figure 6*d*). The top of the chimney appeared to be devoid of other megabenthos, although alvinocaridid shrimps became abundant in the lower parts. As Shisa chimney was not observed in detail and no samples were taken, it is currently not known why only *Branchinotogluma* sp. was abundant there.

The bases of all four chimneys were similar in faunal composition. Most notably, images of the bases are dominated by a high abundance of *Alvinocaris* shrimps and *Munidopsis* squat lobsters (mostly *M. ryukyuensis* with *M. longispinosa* co-occurring at low abundance; figure 6*e*). *Shinkaia crosnieri* is often present too but in low abundance, the same can be said for the shrimp *Lebbeus shinkaiae.* Upon closer examination, a high abundance of *Provanna clathrata* snails was revealed, occasionally together with low abundances of *P. subglabra* and *Bathyacmaea* sp. limpets. Very rarely, one or two *Bathymodiolus aduloides* mussels were seen. Further away from the chimney base, an unidentified demosponge begins to cover sulfide deposits.

Two types of peripheral aggregations were sighted in the Yokosuka site, including relatively focused diffuse flow areas dominated by tubeworm bushes (figure 6*f*) and large, weakly hydrothermally influenced areas dominated by a branching poecilosclerid sponge (figure 6*g*) that often engulfed the tubeworms. With the exception of *Lamellibrachia* being more abundant in tubeworm bush regions where poecilosclerid sponge was not at all present, megafaunal communities observed at these two peripheral regions were generally similar in composition and relative abundance. The mussel *B. aduloides* was common, as well as gastropods *Provanna* spp., *Lepetodrilus nux* and *Bathyacmaea* sp. No other *Bathymodiolus* species were found to inhabit the Yokosuka site, although more thorough exploration in the future may recover other species. The dominance of *B. aduloides* is nevertheless of interest because at all other OT vents it is never present in high abundance [89]), the dominant species being *B. platifrons* and *B. japonicus*. Perhaps, the fact that *B. aduloides* host sulfur-oxidizing endosymbiont (as opposed to methane-oxidizers in *B. platifrons* and *B. japonicus* [89]) contributes to its dominance in the Yokosuka site, but this is largely speculative at this point. *Alaysia* sp. tubeworm was equally abundant in both types of peripheral habitats. The two species of *Alvinocaris* shrimps were also common, although *Shinkaicaris leurokolos* was not seen. An unidentified demosponge was often seen covering hard substrates, more commonly in the sponge field. A single specimen of white-coloured *Lepidonotopodium*? sp. scale worm was seen in a tubeworm bush.

**Figure 10.**
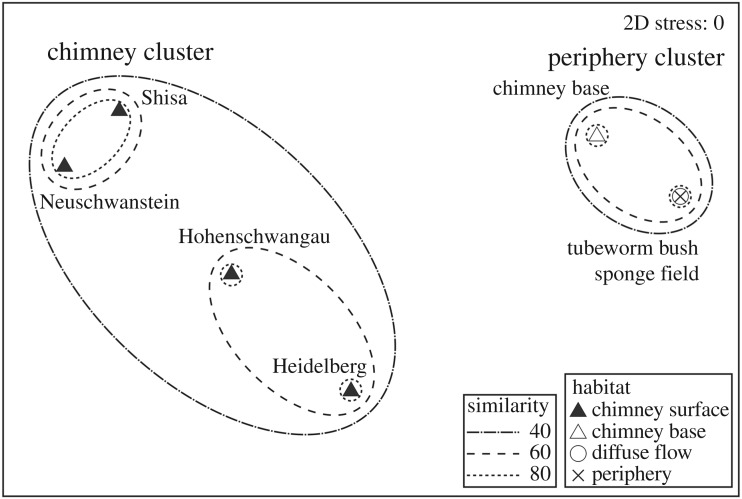
Nonmetric multidimensional scaling (nMDS) plot visualizing similarity in megabenthos composition among the different habitat types identified, based on Jaccard's index of similarity calculated using species presence/absence data. The overlaid contours are based on results from group-average cluster analysis.

The results of similarity analysis of megabenthos composition among the different habitat types identified (figure 10) revealed two highly dissimilar clusters including a ‘chimney’ cluster composed of surfaces of the four active chimneys and a ‘periphery’ cluster composed of chimney bases, tubeworm-dominated diffuse flow and sponge-dominated periphery. All habitats within each cluster had a faunal similarity above 40%. Within the chimney cluster, Neuschwanstein and Shisa were most similar with a faunal similarity of 80%, Hohenschwangau and Heidelberg were rather similar with a similarity of 60%. The reason for detecting the Neuschwanstein–Shisa and Hohenschwangau–Heidelberg pairs may be attributed to the fact that the former lacks ‘peripheral’ taxa such as *S. crosnieri* completely, whereas the latter allows some intrusion of these taxa. Within the periphery cluster, the tubeworm-dominated diffuse flow and sponge-dominated periphery were almost identical in species composition but the chimney base habitat was slightly more dissimilar (60% similarity).

## 4. Conclusion

At a depth of 2190 m the Yokosuka hydrothermal vent site discovered in this study is the deepest OT vent known so far, extending the bathymetric range of hydrothermal activities within the OT by approximately 300 m. The highest fluid temperature recorded in the OT is also extended, as anticipated from the depth, up to 364°C. Neither the depth nor the temperature resulted in large changes in vent fluid compositions at this site compared with other OT sites. However, these fluids were distinct from other OT sites due to the notably high concentrations of H_2_ and CH_4_, probably resulting from inputs of thermogenic volatiles and the preferential venting of vapour-rich phase after subseafloor fluid boiling. Hydrogenotrophic sulfate-reducing bacteria were found to be abundant only on the Neuschwanstein chimney, which exhausts much more H_2_ than the other chimneys analysed. Although the potential of available energy yield from a unit of hydrothermal fluid for chemolithotrophic microbes is high, the total microbial cell density and the overall microbial composition of chimney habitats were not distinct from those known from other OT vent sites. The same is true for megafaunal composition, with species inhabiting the Yokosuka site being in common with those from other OT vents [36,66] despite water depth and aspects of fluid chemistry being distinct.

The preliminary results presented here suggest that there is probably no biogeographic barrier between the 2190 m deep Yokosuka site and other OT vents between 800 and 1650 m deep, and that the Yokosuka site is also part of the well-mixed gene pool of deeper OT vents [21,36]. However, it has been previously indicated that the two shallow sites between 550 and 800 m deep, Minami-Ensei Knoll and Yoron Hole, have different fauna composition compared with other sites [36]. Our early insights from the Yokosuka site implies that although the distribution of animal species may be linked to depth, the constraint is perhaps not water pressure and resulting chemical properties of the vent fluid but instead physical properties of the surrounding seawater, such as density and temperature. For example, a hydrographic barrier for the dispersal may exist at the bottom of thermocline around 700 m [90]. The colder water around the deeper vents may also pose developmental difficulties to larvae of species restricted to shallow vents (and vice versa). As knowledge in biological traits of vent larvae as well as circulation processes in the deep sea are still incomplete, expanding collaborative efforts between physical oceanography and biology (e.g. [20,21]) and detailed analyses of genetic connectivity of shared microbial and faunal taxa among the Yokosuka site and other OT vent sites are required in the future to elucidate whether or not the Yokosuka site is truly well connected with other sites genetically.

According to the bathymetric data available (figure 1) the deepest part of the OT is about 2400 m deep, meaning the Yokosuka site, being 2190 m deep, is in the deepest possible hydrogeographic zone for vents to exist in the OT. Therefore, the apparent lack of biogeographic barrier between the Yokosuka site and other known deeper (greater than 900 m) OT vents, as indicated by the present results, suggests that only two bathymetric biodiversity regions (and therefore gene pools) exist in the OT, with the barrier being around 700–900 m deep. This is an important result in understanding the biogeography and connectivity of vent microbes and fauna both within the OT as well as between OT and other regions such as the Izu–Ogasawara Arc, and also highly relevant to the future conservation and management of vent resources.

## Supplementary Material

Supplementary Table S1

## Supplementary Material

Supplementary Table S2

## Data accessibility

Raw sequence data: GenBank/EMBL/DDBJ accession no. DRA005734 [91]. Other datasets supporting this article have been uploaded as part of the electronic supplementary material.
